# 3,4-Dibromo-2,5-dimethyl-1-phenyl­sulfonyl-1*H*-pyrrole

**DOI:** 10.1107/S1600536811030443

**Published:** 2011-08-02

**Authors:** J. Kanchanadevi, G. Anbalagan, R. Sureshbabu, A. K. Mohanakrishnan, V. Manivannan

**Affiliations:** a Department of Physics, Velammal Institute of Technology, Panchetty, Chennai 601 204, India; b Department of Physics, Presidency College (Autonomous), Chennai 600 005, India; c Department of Organic Chemistry, University of Madras, Guindy campus, Chennai 600 025, India; d Department of Research and Development, PRIST University, Vallam, Thanjavur 613 403, Tamil Nadu, India

## Abstract

In the title compound, C_12_H_11_Br_2_NO_2_S, the dihedral angle between the two rings is 78.79 (12)°. The crystal packing features C—H⋯π inter­actions.

## Related literature

For the biological activity of heterocyclic compounds, see: Ali *et al.* (1989 ▶); Amal Raj *et al.* (2003 ▶). For related structures, see: Seshadri *et al.* (2009 ▶); Gunasekaran *et al.* (2009 ▶).
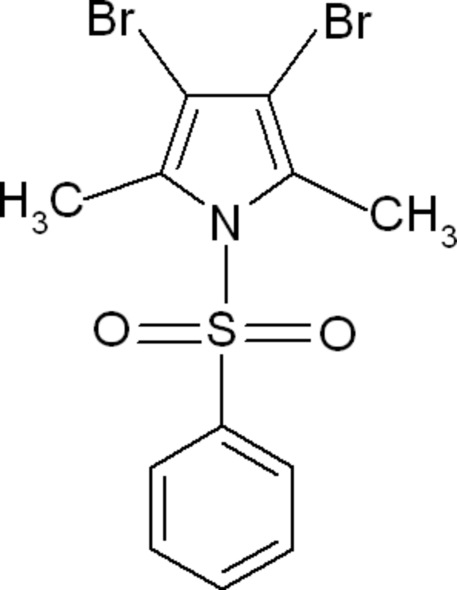


## Experimental

### 

#### Crystal data

C_12_H_11_Br_2_NO_2_S
*M* *_r_* = 393.10
Orthorhombic, 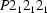

*a* = 6.6248 (4) Å
*b* = 9.7172 (6) Å
*c* = 21.2083 (11) Å
*V* = 1365.27 (14) Å^3^
*Z* = 4
Mo *K*α radiation
μ = 6.08 mm^−1^
*T* = 295 K
0.35 × 0.25 × 0.20 mm

#### Data collection

Bruker Kappa APEXII CCD diffractometer
Absorption correction: multi-scan (*SADABS*; Sheldrick, 1996 ▶) *T*_min_ = 0.945, *T*_max_ = 0.955
9255 measured reflections
3491 independent reflections
2735 reflections with *I* > 2σ(*I*)
*R*_int_ = 0.027

#### Refinement

*R*[*F* ^2^ > 2σ(*F* ^2^)] = 0.034
*wR*(*F* ^2^) = 0.075
*S* = 1.02
3491 reflections
165 parameters
H-atom parameters constrained
Δρ_max_ = 0.47 e Å^−3^
Δρ_min_ = −0.92 e Å^−3^
Absolute structure: Flack (1983 ▶), 1460 Friedel pairs
Flack parameter: 0.010 (10)

### 

Data collection: *APEX2* (Bruker, 2003 ▶); cell refinement: *SAINT* (Bruker, 2003 ▶); data reduction: *SAINT*; program(s) used to solve structure: *SHELXS97* (Sheldrick, 2008 ▶); program(s) used to refine structure: *SHELXL97* (Sheldrick, 2008 ▶); molecular graphics: *PLATON* (Spek, 2009 ▶); software used to prepare material for publication: *SHELXL97*.

## Supplementary Material

Crystal structure: contains datablock(s) global. DOI: 10.1107/S1600536811030443/bt5579sup1.cif

Supplementary material file. DOI: 10.1107/S1600536811030443/bt5579globalsup2.cml

Additional supplementary materials:  crystallographic information; 3D view; checkCIF report

## Figures and Tables

**Table 1:** Hydrogen-bond geometry (Å, °) *Cg*2 is the centroid of the C5–C10 ring.

*D*—H⋯*A*	*D*—H	H⋯*A*	*D*⋯*A*	*D*—H⋯*A*
C12—H12*A*⋯*Cg*2^i^	0.96	2.85	3.545 (7)	130

Symmetry code: (i) 
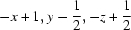
.

**Fig. 1.**
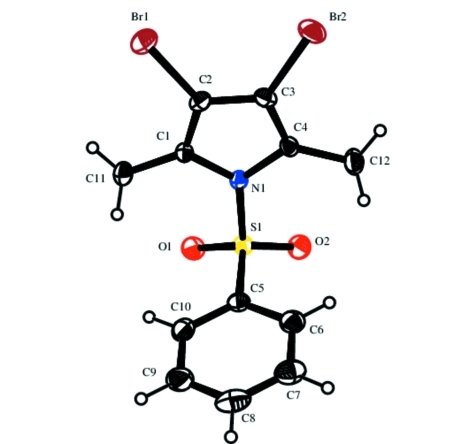
The molecular structure of (I), with atom labels and 30% probability displacement ellipsoids for non-H atoms.

## supplementary crystallographic information

### Comment

Heterocycles, especially five-membered rings, are involved in a wide range of biologically important chemical reactions in living organisms, and therefore they form one of the most important and well investigated classes of organic compounds. They have exhibit antifungal(Amal Raj *et al.*, 2003) and fungicidal (Ali *et al.*, 1989) activity.

The geometric parameters of the title molecule (Fig. 1) agree well with reported similar structure(Seshadri *et al.*, 2009). The phenyl and pyrrole rings inclined at an angle of 78.79 (12) °. The sum of bond angles around N1 [359.6 (3) °] indicates the *sp^2^* hybridization state of atom N1 in the molecule.

The angular disposition of the bonds about the 'S' atom show significant deviation from that a regular tetrahedron, with the largest deviation in O—S—O angle. The widening of angle O1—S1—O2 = 120. 09 (18) ° from the ideal tetrahedral value is the result of the repulsive interactions between the short S=O bonds similar to that observed in other structures (Gunasekaran *et al.*, 2009).

The molecular structure is stabilized by weak intramolecular C—H···O and C—H···Br interactions. The crystal packing is controlled by C—H···π [C12—H12A···*Cg*2(1 - *x*,-1/2 + *y*,1/2 - *z*) distance of 3.545 (7)Å (*Cg*2 is the centroid of the ring defined by the atoms C5—C10)] interaction.

### Experimental

To a solution of tertiary butoxide (1.33 g, 11.85 mmol) and 18-crown -6 (0.15 g, 0.59 mmol) in dry tetrahydrofuran (30 ml), 3,4-dibromo-2,5- dimethyl-1*H*-pyrrole (1.5 g, 5.92 mmol) was added. It was then stirred at room temperature for 30 minutes under nitrogen atmosphere. Then, phenylsulfonyl chloride (0.9 ml, 7.11 mmol) was added through syringe and stirred at the same temperature for 4 h. The reaction mixture was poured to water (100 ml) and extracted with ethylacetate (2 *x* 30 ml). The solvent was removed under reduced pressure.The solid obtained was recrystallized from chloroform to give pure product as a pale brown solid. The yield of the product is 65% and melting point is 515 K.

### Refinement

H atoms were positioned geometrically and refined using riding model with C—H = 0.93Å and *U*_iso_(H) = 1.2Ueq(C) for aromatic C—H, C—H = 0.96Å and *U*_iso_(H) = 1.5Ueq(C) for methyl groups.

### Crystal data

**Table:**

C_12_H_11_Br_2_NO_2_S	*F*(000) = 768
*M**_r_* = 393.10	*D*_x_ = 1.912 Mg m^−^^3^
Orthorhombic, *P*2_1_2_1_2_1_	Mo *K*α radiation, λ = 0.71073 Å
Hall symbol: P 2ac 2ab	Cell parameters from 7415 reflections
*a* = 6.6248 (4) Å	θ = 2.0–28.7°
*b* = 9.7172 (6) Å	µ = 6.08 mm^−^^1^
*c* = 21.2083 (11) Å	*T* = 295 K
*V* = 1365.27 (14) Å^3^	Block, pale brown
*Z* = 4	0.35 × 0.25 × 0.20 mm

### Data collection

**Table:**

Bruker Kappa APEXII CCD diffractometer	3491 independent reflections
Radiation source: fine-focus sealed tube	2735 reflections with *I* > 2σ(*I*)
graphite	*R*_int_ = 0.027
Detector resolution: 0 pixels mm^-1^	θ_max_ = 28.6°, θ_min_ = 1.9°
ω and φ scans	*h* = −8→8
Absorption correction: multi-scan (*SADABS*; Sheldrick, 1996)	*k* = −13→10
*T*_min_ = 0.945, *T*_max_ = 0.955	*l* = −17→28
9255 measured reflections	

### Refinement

**Table:**

Refinement on *F*^2^	Secondary atom site location: difference Fourier map
Least-squares matrix: full	Hydrogen site location: inferred from neighbouring sites
*R*[*F*^2^ > 2σ(*F*^2^)] = 0.034	H-atom parameters constrained
*wR*(*F*^2^) = 0.075	*w* = 1/[σ^2^(*F*_o_^2^) + (0.029*P*)^2^] where *P* = (*F*_o_^2^ + 2*F*_c_^2^)/3
*S* = 1.02	(Δ/σ)_max_ = 0.001
3491 reflections	Δρ_max_ = 0.47 e Å^−^^3^
165 parameters	Δρ_min_ = −0.92 e Å^−^^3^
0 restraints	Absolute structure: Flack (1983), 1460 Friedel pairs
Primary atom site location: structure-invariant direct methods	Flack parameter: 0.010 (10)

### Fractional atomic coordinates and isotropic or equivalent isotropic displacement parameters (Å^2^)

**Table:**

	*x*	*y*	*z*	*U*_iso_*/*U*_eq_	
C1	0.1597 (5)	0.2073 (3)	0.41513 (14)	0.0293 (7)	
C2	0.1708 (5)	0.0859 (3)	0.44645 (14)	0.0327 (8)	
C3	0.3469 (6)	0.0151 (3)	0.42718 (15)	0.0329 (7)	
C4	0.4461 (5)	0.0895 (3)	0.38337 (14)	0.0298 (7)	
C5	0.2313 (6)	0.2831 (3)	0.25488 (15)	0.0327 (8)	
C6	0.3161 (7)	0.1922 (4)	0.21200 (17)	0.0469 (10)	
H6	0.4434	0.1548	0.2190	0.056*	
C7	0.2079 (8)	0.1584 (4)	0.15883 (18)	0.0549 (11)	
H7	0.2633	0.0990	0.1291	0.066*	
C8	0.0186 (8)	0.2121 (4)	0.14939 (18)	0.0596 (13)	
H8	−0.0538	0.1877	0.1135	0.072*	
C9	−0.0642 (7)	0.3002 (5)	0.19171 (19)	0.0610 (12)	
H9	−0.1935	0.3344	0.1850	0.073*	
C10	0.0435 (6)	0.3399 (5)	0.24533 (17)	0.0499 (10)	
H10	−0.0102	0.4030	0.2738	0.060*	
C11	0.0040 (6)	0.3164 (4)	0.42098 (18)	0.0449 (9)	
H11A	−0.0988	0.2870	0.4499	0.067*	
H11B	−0.0552	0.3336	0.3804	0.067*	
H11C	0.0655	0.3993	0.4364	0.067*	
C12	0.6350 (6)	0.0542 (4)	0.34928 (18)	0.0474 (10)	
H12A	0.6855	−0.0324	0.3643	0.071*	
H12B	0.7340	0.1246	0.3565	0.071*	
H12C	0.6077	0.0476	0.3049	0.071*	
N1	0.3311 (4)	0.2116 (3)	0.37564 (12)	0.0285 (6)	
O1	0.2928 (4)	0.4577 (2)	0.34533 (11)	0.0435 (6)	
O2	0.5824 (4)	0.3247 (3)	0.30554 (12)	0.0448 (6)	
S1	0.37395 (13)	0.33329 (9)	0.32099 (4)	0.03095 (19)	
Br1	−0.00952 (7)	0.02664 (5)	0.50804 (2)	0.05823 (14)	
Br2	0.43075 (7)	−0.15500 (4)	0.45930 (2)	0.05195 (13)	

### Atomic displacement parameters (Å^2^)

**Table:**

	*U*^11^	*U*^22^	*U*^33^	*U*^12^	*U*^13^	*U*^23^
C1	0.0269 (18)	0.0320 (17)	0.0290 (17)	0.0039 (15)	0.0017 (15)	−0.0010 (14)
C2	0.0326 (19)	0.0388 (19)	0.0267 (18)	−0.0042 (16)	0.0028 (15)	−0.0001 (14)
C3	0.0411 (19)	0.0277 (16)	0.0299 (17)	0.0031 (15)	−0.0065 (15)	0.0011 (13)
C4	0.032 (2)	0.0269 (15)	0.0301 (17)	0.0053 (15)	−0.0025 (15)	−0.0039 (13)
C5	0.036 (2)	0.0324 (17)	0.0294 (18)	−0.0045 (16)	−0.0004 (15)	0.0039 (14)
C6	0.063 (3)	0.037 (2)	0.041 (2)	0.006 (2)	0.003 (2)	0.0050 (17)
C7	0.081 (3)	0.042 (2)	0.041 (2)	−0.006 (3)	−0.003 (2)	−0.0039 (19)
C8	0.075 (4)	0.068 (3)	0.036 (2)	−0.025 (3)	−0.008 (2)	0.005 (2)
C9	0.040 (2)	0.100 (4)	0.043 (2)	−0.002 (2)	−0.006 (2)	0.008 (2)
C10	0.039 (2)	0.075 (3)	0.036 (2)	0.004 (2)	0.0026 (17)	−0.0014 (18)
C11	0.046 (2)	0.045 (2)	0.044 (2)	0.014 (2)	0.011 (2)	−0.0015 (16)
C12	0.043 (2)	0.040 (2)	0.059 (2)	0.0122 (19)	0.012 (2)	0.0013 (18)
N1	0.0301 (16)	0.0276 (13)	0.0279 (13)	0.0044 (12)	0.0036 (13)	−0.0001 (12)
O1	0.0573 (17)	0.0265 (12)	0.0466 (15)	0.0032 (13)	0.0010 (13)	−0.0011 (11)
O2	0.0325 (13)	0.0490 (15)	0.0528 (15)	−0.0089 (13)	0.0050 (12)	0.0076 (12)
S1	0.0317 (4)	0.0273 (4)	0.0339 (4)	−0.0018 (4)	0.0008 (4)	0.0021 (3)
Br1	0.0557 (3)	0.0637 (3)	0.0552 (2)	0.0000 (2)	0.0204 (2)	0.0186 (2)
Br2	0.0661 (3)	0.03477 (19)	0.0549 (2)	0.0103 (2)	−0.0033 (2)	0.01168 (17)

### Geometric parameters (Å, °)

**Table:**

C1—C2	1.356 (4)	C7—H7	0.9300
C1—N1	1.412 (4)	C8—C9	1.356 (6)
C1—C11	1.484 (5)	C8—H8	0.9300
C2—C3	1.415 (5)	C9—C10	1.397 (5)
C2—Br1	1.861 (3)	C9—H9	0.9300
C3—C4	1.348 (5)	C10—H10	0.9300
C3—Br2	1.872 (3)	C11—H11A	0.9600
C4—N1	1.420 (4)	C11—H11B	0.9600
C4—C12	1.486 (5)	C11—H11C	0.9600
C5—C10	1.377 (5)	C12—H12A	0.9600
C5—C6	1.386 (5)	C12—H12B	0.9600
C5—S1	1.760 (3)	C12—H12C	0.9600
C6—C7	1.376 (5)	N1—S1	1.680 (3)
C6—H6	0.9300	O1—S1	1.420 (2)
C7—C8	1.373 (7)	O2—S1	1.422 (3)
			
C2—C1—N1	105.8 (3)	C10—C9—H9	119.8
C2—C1—C11	128.2 (3)	C5—C10—C9	118.1 (4)
N1—C1—C11	126.0 (3)	C5—C10—H10	121.0
C1—C2—C3	109.1 (3)	C9—C10—H10	121.0
C1—C2—Br1	125.3 (3)	C1—C11—H11A	109.5
C3—C2—Br1	125.5 (3)	C1—C11—H11B	109.5
C4—C3—C2	109.9 (3)	H11A—C11—H11B	109.5
C4—C3—Br2	125.4 (3)	C1—C11—H11C	109.5
C2—C3—Br2	124.7 (3)	H11A—C11—H11C	109.5
C3—C4—N1	105.4 (3)	H11B—C11—H11C	109.5
C3—C4—C12	128.5 (3)	C4—C12—H12A	109.5
N1—C4—C12	126.1 (3)	C4—C12—H12B	109.5
C10—C5—C6	121.7 (4)	H12A—C12—H12B	109.5
C10—C5—S1	119.4 (3)	C4—C12—H12C	109.5
C6—C5—S1	118.8 (3)	H12A—C12—H12C	109.5
C7—C6—C5	118.6 (4)	H12B—C12—H12C	109.5
C7—C6—H6	120.7	C1—N1—C4	109.8 (3)
C5—C6—H6	120.7	C1—N1—S1	124.5 (2)
C8—C7—C6	120.3 (4)	C4—N1—S1	125.3 (2)
C8—C7—H7	119.8	O1—S1—O2	120.09 (18)
C6—C7—H7	119.8	O1—S1—N1	106.52 (14)
C9—C8—C7	120.9 (4)	O2—S1—N1	106.36 (15)
C9—C8—H8	119.6	O1—S1—C5	108.82 (17)
C7—C8—H8	119.6	O2—S1—C5	108.76 (17)
C8—C9—C10	120.4 (4)	N1—S1—C5	105.30 (15)
C8—C9—H9	119.8		
			
N1—C1—C2—C3	0.2 (4)	C2—C1—N1—C4	0.4 (3)
C11—C1—C2—C3	−178.7 (3)	C11—C1—N1—C4	179.3 (3)
N1—C1—C2—Br1	177.2 (2)	C2—C1—N1—S1	172.8 (2)
C11—C1—C2—Br1	−1.7 (5)	C11—C1—N1—S1	−8.3 (5)
C1—C2—C3—C4	−0.8 (4)	C3—C4—N1—C1	−0.9 (3)
Br1—C2—C3—C4	−177.8 (2)	C12—C4—N1—C1	178.9 (3)
C1—C2—C3—Br2	178.1 (2)	C3—C4—N1—S1	−173.2 (2)
Br1—C2—C3—Br2	1.1 (4)	C12—C4—N1—S1	6.5 (5)
C2—C3—C4—N1	1.0 (4)	C1—N1—S1—O1	33.9 (3)
Br2—C3—C4—N1	−177.9 (2)	C4—N1—S1—O1	−154.8 (3)
C2—C3—C4—C12	−178.7 (3)	C1—N1—S1—O2	163.1 (3)
Br2—C3—C4—C12	2.4 (5)	C4—N1—S1—O2	−25.6 (3)
C10—C5—C6—C7	−0.2 (6)	C1—N1—S1—C5	−81.6 (3)
S1—C5—C6—C7	−176.9 (3)	C4—N1—S1—C5	89.7 (3)
C5—C6—C7—C8	−1.2 (6)	C10—C5—S1—O1	−16.9 (3)
C6—C7—C8—C9	0.8 (6)	C6—C5—S1—O1	159.8 (3)
C7—C8—C9—C10	1.1 (7)	C10—C5—S1—O2	−149.4 (3)
C6—C5—C10—C9	2.0 (6)	C6—C5—S1—O2	27.4 (3)
S1—C5—C10—C9	178.6 (3)	C10—C5—S1—N1	96.9 (3)
C8—C9—C10—C5	−2.4 (7)	C6—C5—S1—N1	−86.3 (3)

### Hydrogen-bond geometry (Å, °)

**Table:**

Cg2 is the centroid of the C5–C10 ring.

**Table:**

*D*—H···*A*	*D*—H	H···*A*	*D*···*A*	*D*—H···*A*
C10—H10···O1	0.93	2.57	2.922 (5)	103.
C11—H11A···Br1	0.96	2.88	3.368 (4)	113.
C11—H11C···O1	0.96	2.51	2.849 (5)	100.
C12—H12A···Br2	0.96	2.88	3.378 (4)	113.
C12—H12B···O2	0.96	2.44	2.809 (5)	102.
C12—H12A···Cg2^i^	0.96	2.85	3.545 (7)	130.

Symmetry codes: (i) −*x*+1, *y*−1/2, −*z*+1/2.

## References

[bb1] Ali, R., Misra, B. & Nizamuddin, M. (1989). *Indian J. Chem. Sect. B*, **28**, 526–528.

[bb2] Amal Raj, A., Raghunathan, R., Sridevikumari, M. R. & Raman, N. (2003). *Bioorg. Med. Chem.* **11**, 407–419. 10.1016/s0968-0896(02)00439-x 12517436

[bb3] Bruker (2003). *APEX2* and *SAINT* Bruker AXS Inc., Madison, Wisconsin, USA.

[bb4] Flack, H. D. (1983). *Acta Cryst.* A**39**, 876–881.

[bb5] Gunasekaran, B., Sureshbabu, R., Mohanakrishnan, A. K., Chakkaravarthi, G. & Manivannan, V. (2009). *Acta Cryst.* E**65**, o2069. 10.1107/S1600536809029985 PMC2970018 21577492

[bb6] Seshadri, P. R., Balakrishnan, B., Ilangovan, K., Sureshbabu, R. & Mohanakrishnan, A. K. (2009). *Acta Cryst.* E**65**, o531. 10.1107/S1600536809004425 PMC2968504 21582192

[bb7] Sheldrick, G. M. (1996). *SADABS*, University of Göttingen, Germany.

[bb8] Sheldrick, G. M. (2008). *Acta Cryst.* A**64**, 112–122. 10.1107/S0108767307043930 18156677

[bb9] Spek, A. L. (2009). *Acta Cryst.* D**65**, 148–155. 10.1107/S090744490804362X PMC2631630 19171970

